# Sexuality in Postmenopausal Women with Genital Prolapse

**DOI:** 10.3390/jcm12196290

**Published:** 2023-09-29

**Authors:** Ana Cristina Fernández Rísquez, Antonio Carballo García, Jesús Joaquín Hijona Elósegui, Nicolás Mendoza Ladrón de Guevara, Jesús Carlos Presa Lorite

**Affiliations:** 1 Obstetrics and Gynecology Department, University Hospital of Jaén, CP 23007 Jaén, Spain; anacfr88@gmail.com (A.C.F.R.); jesushijona@gmail.com (J.J.H.E.); jpresalorite@hotmail.com (J.C.P.L.); 2 Department of Obstetrics and Gynecology, University of Granada, CP 18071 Granada, Spain; nicomendozaladron@gmail.com

**Keywords:** prolapse, sexual dysfunction, sexuality, menopause

## Abstract

Background: One of the most common complaints among menopausal women concerns changes in sexual function. This is attributed to various factors, including anatomical defects in the genital tract, with pelvic organ prolapse (POP) being one of the most prevalent problems affecting women during this stage of their lives. Additionally, symptoms resulting from gonadal hypofunction can also contribute to the development of sexual dysfunction during menopause. This research aimed to explore the way in which postmenopausal patients with POP experienced their sexuality in our setting. Methods: To achieve the proposed objective, we conducted a descriptive, cross-sectional study involving a total of 133 postmenopausal women with POP. Results: The results of our series are consistent with the scarce literature available in our setting and suggest a high rate of sexual dysfunction in postmenopausal patients with POP. Conclusions: We can conclude that POP is associated with the presence of female sexual dysfunction.

## 1. Introduction

Due to the increase in life expectancy, the number of postmenopausal women has increased considerably and with it the number of pathologies associated with this period of life, such as pelvic organ prolapse (POP). Although physiological, this is a condition that also predisposes to certain pathologies, and its symptoms depend on physiological, psychological, and socio-cultural factors [1].

The presence of POP is a pathology that has been increasing and that causes important limitations in the quality of life of women, as well as requiring numerous medical and surgical actions for its correction and prevention. For all these reasons, the interest in this area of research and its potential usefulness in routine clinical practice lies in being able to identify patients with prolapse [2].

POP is a variety of hernia of the urogenital hiatus and can generate a multisystemic pathology, thus requiring a multidisciplinary approach. It constitutes an important part of pelvic floor dysfunction and can be associated with various processes: urinary dysfunction, different degrees of prolapse, defecatory dysfunction, chronic pelvic pain, and, of course, sexual dysfunction. Depending on the organ prolapsed, we can speak of cystocele, urethrocele, uterine prolapse, dome prolapse in hysterectomized women, and enterocele or rectocele. It is also one of the most common indications for gynecological surgery, with 11.8% of women being operated on for POP in their lifetime, which accounts for up to 30% of major gynecological surgeries in our field [3].

The exact prevalence is difficult to determine and is not known exactly, due to the lack of consensus on an exact definition of this pathology and the fact that patients do not usually come to the clinic for this reason [4]. It is a multifactorial pathology and develops gradually over the years, and although many risk factors have been proposed, the relative importance of each factor is unknown [3].

Genital prolapse in its initial stages is usually asymptomatic, being a casual finding in a gynecological examination. The main symptom of POP is the sensation of a genital lump; this symptom is independently associated with the severity of the prolapse. Other symptoms are urinary (stress and urge urinary incontinence, repeated infections, urinary retention), gastrointestinal (anal incontinence of gas and feces), sexual dysfunction (dyspareunia, decreased libido, problems reaching orgasm), and pelvic pain in the lumbosacral region, but there is little evidence to suggest a direct relationship [5,6,7]. Diagnosis is primarily clinical by thorough physical examination. 

Furthermore, another of the most common complaints among menopausal women is changes in their sexual function, due, among other factors, to the frequent presence of anatomical defects in the genital tract, which, combined with the symptoms derived from gonadal hypofunction, can be a determining factor in the appearance of sexual dysfunction during menopause [1].

The present research aimed to explore the way in which menopausal patients with POP experience their sexuality in our setting.

The aim of this study was to assess the impact of pelvic organ prolapse on sexual function in menopausal women.

## 2. Materials and Methods

### 2.1. Design

To achieve the proposed objective, we set up a descriptive, prospective, cross-sectional study, in which sexual function was assessed in postmenopausal women with any type of POP by means of a self-compliance questionnaire, at the University Hospital of Jaén, over a period of 14 months.

### 2.2. Study Population and Sample

A total of 132 postmenopausal women with POP who attended the outpatient clinic of the University Hospital of Jaén between April 2019 and July 2020 and who were representative of the population attending our center were included. The volunteers agreed to participate in the study and signed an informed consent form. This study was approved by the Research Ethics Committee of the Province of Jaén prior to its initiation with a favorable report. 

The sample size was calculated based on the current reported prevalence of POP and sexual dysfunction in the Spanish menopausal female population, applying the Rogan and Gladen formula for estimating population prevalence from the results of a screening test. The data were collected, cleaned, and analyzed using the IBM SPSS Statistics 28 statistical package version 21.

### 2.3. Methodology

The analysis of the epidemiological data was carried out by creating a database and classifying the different findings of the patients for the different variables. The responses were then coded in the database for interpretation by means of statistical analysis, and the results were obtained.

To classify the degree of POP, a basic gynecological examination was carried out with the patient in the gynecological position and through inspection with a speculum and the Valsalva maneuver, documenting the degree of POP, and classifying it using the simplified POP-Q scale, a 5-stage ordinal system with a faster application in daily routine activity and with the same validity as the POP-Q classification.

**Figure 1:**
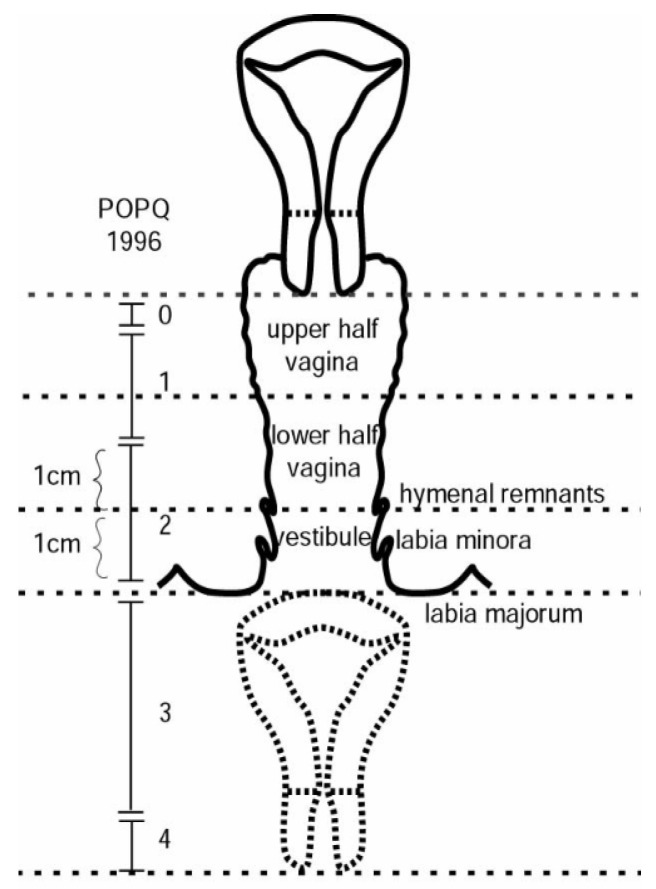
Simplified POP-Q scale [8].

**Figure 2:**
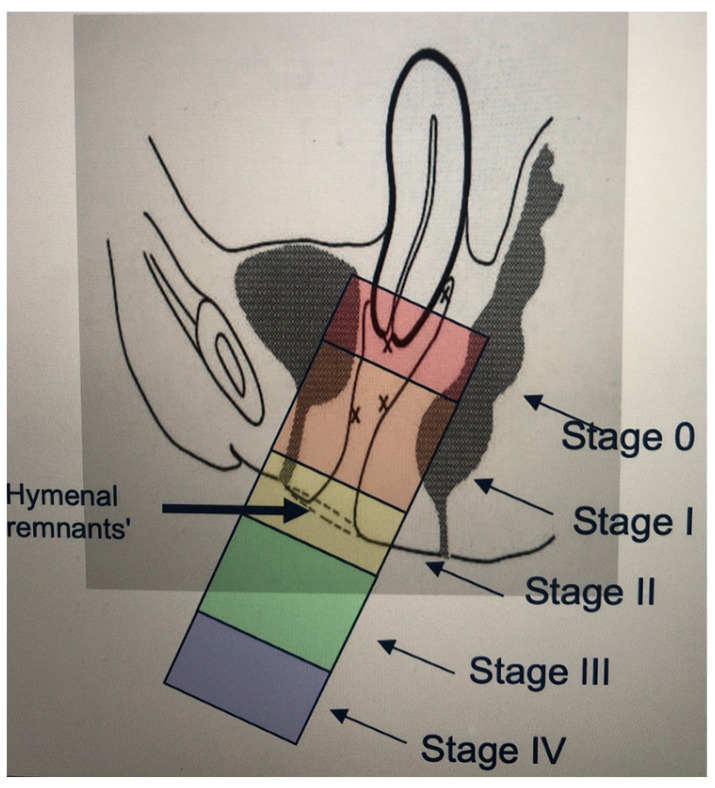
Simplified POP-Q scale [9].

- Stage 0: No evidence of prolapse.
- Stage I: The steepest point of prolapse is more than 1 cm above the remnants of the hymen.
- Stage II: The steepest point of prolapse is in the area between 1 cm above and 1 cm below the hymenal remnants.
- Stage III: The steepest point of prolapse is more than 1 cm below the remnants of the hymen.
- Stage IV: This is total prolapse, in which the vaginal mucosa is completely everted (Figure 1 and Figure 2).

Sexual activity and function were assessed in all participants using the female sexual function (FSM) questionnaire. In addition to assessing women’s sexual response, this questionnaire collects important descriptive aspects about their sexual activity with their partner: anticipatory anxiety, initiative, and confidence [10].

The study was approved by the Research Ethics Committee of the province of Jaén and was carried out according to the regulations established in the law 14/2007 of 3 July on biomedical research, following the precepts included in the Belmont report and the declaration of Helsinki (updated in the Brazilian assembly in 2013) for biomedical research.

### 2.4. Selection Criteria

All women who met the following criteria were included in the study:

- Out-patients consulting a gynecologist
- Women over 50
- Documented menopausal status
- Consented to participate in the study.

On the other hand, exclusion criteria for participation in the study were considered to be patients with early menopause and those under treatment with drugs simulating primary ovarian failure.

For the collection of epidemiological data and inclusion and exclusion criteria, a thorough review of all the participants’ digital medical records was carried out at the time of the first consultation, during which all patients completed the FSM evaluator questionnaire, validated in Spain. Subsequently, a self-completion questionnaire was used to collect information on known risk factors for POP, as well as sociodemographic factors and gynecological and obstetric history.

### 2.5. Statistical Analysis

The data collected on the dependent and independent variables mentioned in this study were recorded in an anonymized database constructed for this purpose and statistically processed using SPSS v21 and R 4.2.1 statistical software.

Quantitative variables were represented by means of the mean and deviation in the absence of normality, and nominal variables by frequency and percentage. A normality study was carried out using a Shapiro–Wilk test.

A descriptive statistical analysis was carried out for each of the variables in the database. For this purpose, a table of frequencies (number of cases and percentage) was presented for the variables studied, and several bar graphs were made as a graphical representation.

## 3. Results

The epidemiological characteristics of the study population are presented in Table 1. Of these, only one loss due to illness or relocation of place of residence was recorded.

The mean age of the participants included in the study was 64.47 ± 8.58 years. For the calculations, the participants were grouped into low grade (I-II) and high grade POP (III-IV), with 49.6% (n = 66) presenting grade I-II POP and 50.4% (n = 67) grade III-IV POP.

The FSM questionnaire is a method for diagnosing sexual dysfunction that assesses five domains: anticipatory anxiety, orgasm, sexual initiative, sexual satisfaction, and problems with vaginal penetration. Table 2 shows the results obtained in our series.

A total of 132 patients with a mean age of 64.47 years (range 51–87) were included. In total, almost half of the participants never felt fear or anxiety about the possibility of sexual intercourse, and women who almost always/always feel fear or anxiety about the possibility of sexual intercourse accounted for 20% of the total sample. If this variable is converted to a scale of 1 to 5 (1 = never and 5 = almost always/always), the mean for our population was 1.21 ± 0.15, meaning that they rarely felt fear or uneasiness (Table 3).

Second, in relation to orgasm, there was a higher percentage of women who never or rarely reach orgasm (51.9%) and only 21.8% who almost always/always reach orgasm. If this variable is converted into a scale from 1 to 5 (1 = never and 5 = almost always/always), the mean for the population was 1.63 ± 1.56. This means that on average the women in our study never or rarely reach orgasm (Table 4).

On the other hand, when asked how often it is the woman who takes the initial steps to provoke the sexual encounter, more than 2/3 never or rarely initiate it (80.9%). In contrast, only 1.8% do it almost always/always. If this variable is converted into a scale from 1 to 5 (1 = never and 5 = almost always/always), the mean for the population was 0.63 ± 0.97. Approximating to the nearest class, on average, our population never takes the initial steps to provoke a sexual encounter (Table 5).

Regarding the degree of satisfaction with their sex life in recent weeks, almost half of the population was neither satisfied nor dissatisfied (43%), while 24.3% were fairly or very dissatisfied. If this variable is converted into a scale from 1 to 5 (1 = very dissatisfied and 5 = very satisfied), the mean for the population was 3.02 ± 1.22, i.e., on average, the women were neither satisfied nor dissatisfied with their sex life (Table 6).

Finally, when asked whether they avoid sexual intercourse because of vaginal lumps, one third of the sample said that they never or rarely avoid sexual intercourse (37.4%), compared to 42% who always or frequently avoid it. If this variable is converted into a scale of 1 to 5 (1 = never and 5 = always), the population mean was 2.92 ± 1.57 (Table 7).

In conclusion, half of the women evaluated never feel afraid of the possibility of sexual intercourse and 51.9% have anorgasmia. In general, women with POP and menopausal women never take the first steps to initiate sexual intercourse and almost half of them reported feeling neither satisfied nor dissatisfied with their sex life; finally, about half of the participants avoid sexual intercourse because of lumps in the vagina (Table 8).

**Figure 3:**
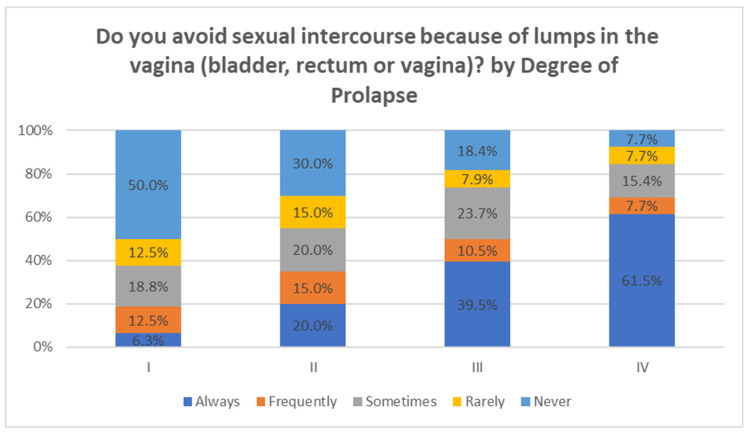
Relationship of POP and satisfaction with sex life.

**Figure 4:**
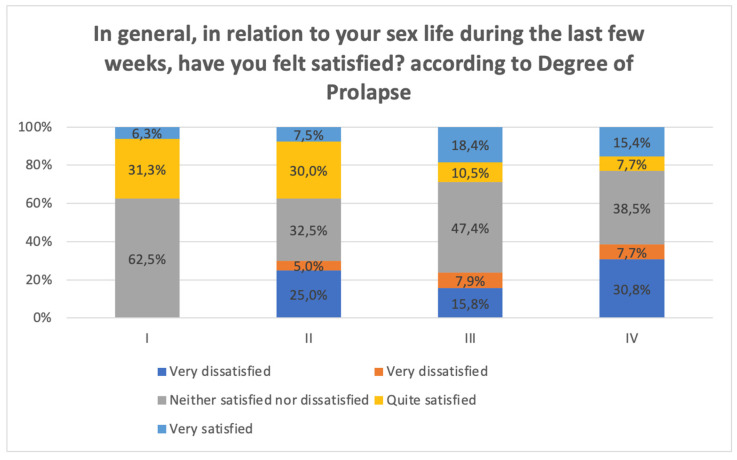
Relationship between POP and avoidance of sex.

Finally, as can be seen in Figure 3 and Figure 4, there was an inverse relationship between the degree of prolapse and sexual satisfaction, with avoidance of penetrative intercourse being more frequent the greater the degree of prolapse. It is worth noting that almost half felt indifferent about their sex life.

## 4. Discussion

Genital prolapse is defined as the descent or displacement of the pelvic organs as a result of the failure of the supporting structures and is a condition with specific signs and symptoms that impairs normal function and reduces the quality of life of women [2]. As it is a very common problem, its prevalence is estimated to be between 4% and 76%, assuming that the prevalence is high and probably underestimated. The Women’s Health Initiative (WHI) study describes that 41% of non-hysterectomized women and 38% of hysterectomized women had some form of prolapse. A study in the US population estimated that 41% of women aged 50–79 years had some degree of genital prolapse, with an estimated prevalence of one or more pelvic floor disorders of 23.7% and a 10% risk of undergoing surgery [4]. Although data are limited, studies show that the prevalence of pelvic organ prolapse increases with age; thus, the prevalence of pelvic organ prolapse will undoubtedly increase in the coming years.

The current classification of prolapse is based on the Pelvic Organ Prolapse Quantification System (POP-Q) scale: Pelvic Organ Prolapse Quantification System (POP-Q) proposed by the International Continence Society-International Urogynecological Association (ICS-IUGA) [11]. This system is based on the measurement of several clearly defined anatomical landmarks, which are measured during the Valsalva maneuver at the hymen and should reflect the maximum protrusion [2]. This system can be perceived as complex, and for this reason the ICSI devised a simplified classification system that retains the ordinal stages but simplifies the terminology and reduces the number of points measured (simplified POP-Q) [8]. For this reason, this was the classification used in this study, because of its greater simplicity and reproducibility.

On the other hand, sexuality is a cardinal aspect of women’s quality of life and is a very illuminating reflection of their level of physical, psychological, and social well-being [9].

Female sexual dysfunction is defined as a disorder, at any stage of the sexual response, that is capable of generating personal stress and having a negative impact on quality of life and interpersonal relationships [3]. Their etiologies are multifactorial and their actual incidence is currently unknown [12]. They are often a taboo subject in our society at different levels and, unsurprisingly, also during clinical interviews [13], which makes self-completion questionnaires such as the FSM an emerging tool to facilitate diagnosis, as they facilitate data collection by allowing the patient to fill them in privately and anonymously [14]. The use of questionnaires in the pelvic floor consultation and especially in the sexuality consultation has been confirmed as a fundamental tool for both diagnosis and when establishing a therapy or medical treatment. Despite the facilities provided by this type of questionnaire, a significant number of patients do not wish to answer. It should also be noted that this type of questionnaire does not specifically cover dysfunctions derived from male pathology, which means that a significant number of patients do not answer or inform us in the survey comments that the absence of sexual relations is due to a problem or pathology of their partner. Currently, in sexuality consultations, a global study of the couple is being carried out, studying both members of the couple and making a diagnosis independently of each of the members and then jointly. This is why it is necessary to specifically train professionals in sexuality, in order to improve the diagnosis and treatment of these pathologies [10,14].

The system for the classification of sexual dysfunctions in women has evolved from a mechanical and linear conception, to a simpler one where the various planes overlap. Today, this tendency to oversimplify the phenomenon of sexual dysfunction continues to be a source of criticism and controversy among professionals dedicated to the integral care of women’s health [15].

It is estimated that between 40 and 80% of menopausal women present dysfunctions in the area of sexuality [16]. The decrease in estrogen levels leads to important changes in the anatomy and function of the genitalia, producing different symptoms that can affect daily life and ultimately have a negative impact on the sexual sphere. On the other hand, pelvic floor pathology, which affects one in three adult women, can also negatively influence the sexual activity of the patient suffering from it [13]. 

Based on the above and the results observed in our study, it can be hypothesized that the presence of pelvic floor pathology in menopausal women may significantly impair their sexual health; however, to the best of our knowledge, there has been very little literature published on this subject in our field. There are tools specifically designed to assess sexual function in women in general and also in women affected by POP, urinary incontinence, and/or fecal incontinence. The best known of the latter is the PISQ-IR, but its variables do not include the patient’s pre- or post-menopausal status [17]. 

The results obtained in our series are consistent with the scarce literature available on the subject in our field [1,18] and with the fact that having POP could have a negative influence on how women cope with sexual relations, how they experience them, and, in particular, how satisfactory they are. This could be due to the presence of a lump in the vagina, either because of an anatomical and functional alteration that has occurred, or because of the patient’s own self-perception. In any case, we currently lack validated tools for clinical application in the menopausal population with pelvic organ prolapse. We therefore consider it essential to undertake new lines of research on this particular subject [1].

Finally, as we have observed, POP is associated with the presence of female sexual dysfunction, with a direct relationship between the degree of prolapse and the intensity of sexual dysfunction. On the other hand, the assessment of sexual function by means of specific questionnaires facilitates the identification and treatment of sexual dysfunctions associated with pelvic floor pathology. 

The limitations of our study include the fact that it was a cross-sectional, descriptive study, and therefore there was no control group of women without POP; moreover, the sample was perhaps too small. In addition to increasing the sample, it would be necessary to create an experimental study to improve the quality of the study and draw better conclusions. 

## 5. Conclusions

Our findings demonstrate a direct relationship between the severity of POP and the intensity of sexual dysfunction experienced by postmenopausal patients. Moreover, the utilization of dedicated questionnaires to assess sexual function would be of value for identifying and treating sexual dysfunction linked to pelvic floor pathology. 

## Figures and Tables

**Table 1:** Epidemiological characteristics.

Age in Years, Mean (SD)	64.47 (8.58)
Weight in kg, mean (SD)	67.63 (9.44)
Height in cm, mean (SD)	158.87 (5.99)
BMI in Kg/cm^2^ mean (SD)	26.85 (3.89)
*Underweight n (%)*	2 (1.5)
*Normal weight n (%)*	45 (33.8)
*Overweight n (%)*	63 (47.4)
*Obesity n (%)*	23 (17.3)
Marital status, n (%)	
*Married*	108 (81.2)
*Single*	7 (5.3)
*Widow*	18 (13.5)
Area of residence n (%)	
*Rural*	42 (31.6)
*Urban*	91 (68.4)
Level of education, n (%)	
*Primary education*	74 (55.6)
*Secondary education*	18 (13.5)
*Higher education*	11 (8.3)
*No education*	30 (22.6)
Physical work activity, n (%)	87 (65.4)
Tobacco use, n (%)	
*<10 cigarettes/day*	12 (9)
*>10 cigarettes/day*	2 (1.5)
*Non-smoker*	119 (89.5)
Alcohol consumption, n (%)	32 (24.1)
Pregnancies, mean (SD)	3.25 (1.73)
Number of births, mean (SD)	2.93 (1.34)
Increase in abdominal pressure n (%)	43 (32.3)

SD: standard deviation.

**Table 2:** Summary of results.

	Never	Rarely	Sometimes	Often	Almost Always/ Always	Ns/Nc
During the last few weeks, when faced with the idea or possibility of sexual activity, have you felt fear, uneasiness, anxiety…, n (%)	63 (47.4)	11 (8.3)	8 (6.0)	6 (4.5)	22 (16.5)	23 (17.3)
During the last few weeks, have you reached orgasm when you have engaged in sexual activity, with or without penetration? n (%)	40 (30.1)	17 (12.8)	21 (15.8)	8 (6.0)	24 (18.0)	23 (17.3)
In the last few weeks, how many times have you taken the initial steps to provoke a sexual encounter with another person, n (%)?	70 (52.6)	19 (14.3)	15 (11.3)	4 (3.0)	2 (1.5)	23 (17.3)
	Very dissatisfied	Quite dissatisfied	Neither satisfied nor dissatisfied	Quite satisfied	Very satisfied	Ns/Nc
In general, in relation to your sex life during the last few weeks, have you felt satisfied, n(%)?	20 (15.0)	6 (4.5)	46 (34.6)	22 (16.5)	13 (9.8)	26 (19.5)
	Always	Frequently	Sometimes	Rarely	Never	Ns/Nc
Do you avoid sexual intercourse because of lumps in the vagina (bladder, rectum or vagina) ?, n (%)	32 (24.1)	13 (9.8)	22 (16.5)	12 (9.0)	28 (21.1)	26 (19.5)

Ns/Nc: don’t know/no answer.

**Table 3:** FSM Questionnaire.

1. During the Last Few Weeks, at the Idea or Possibility of Sexual Activity, Have You Felt Fear, Uneasiness, Anxiety…?					
		Frequency	Percentage	Percentage Valid	Cumulative Percentage
Valid	Never	63	47.4	57.3	57.3
	Rarely	11	8.3	10.0	67.3
	Sometimes	8	6.0	7.3	74.5
	Often	6	4.5	5.5	80.0
	Almost always/Always	22	16.5	20.0	100.0
	Total	110	82.7	100.0	
Lost	Lost	23	17.3		
Total		133	100.0		

**Table 4:** FSM Questionnaire.

2. During the Last Few Weeks, Have You Reached Orgasm when You Have Engaged in Sexual Activity, with or without Penetration?					
		Frequency	Percentage	Percentage Valid	Cumulative Percentage
Valid	Never	40	30.1	36.4	36.4
	Rarely	17	12.8	15.5	51.8
	Sometimes	21	15.8	19.1	70.9
	Often	8	6.0	7.3	78.2
	Almost always/Always	24	18.0	21.8	100.0
	Total	110	82.7	100.0	
Lost	Lost	23	17.3		
Total		133	100.0		

**Table 5:** FSM Questionnaire.

3. In the Last Few Weeks, How Many Times Have You Been the One Who Has Taken the Initial Steps to Provoke a Sexual Encounter with Another Person?					
		Frequency	Percentage	Percentage Valid	Cumulative Percentage
Valid	Never	70	52.6	63.6	63.6
	Rarely	19	14.3	17.3	80.9
	Sometimes	15	11.3	13.6	94.5
	Often	4	3.0	3.6	98.2
	Almost always/Always	2	1.5	1.8	100.0
	Total	110	82.7	100.0	
Lost	Lost	23	17.3		
Total		133	100.0		

**Table 6:** FSM Questionnaire.

4. In general, in Relation to Your Sex Life During the Last Few Weeks, Have You Felt Satisfied?					
		Frequency	Percentage	Percentage Valid	Cumulative Percentage
Valid	Very dissatisfied	20	15.0	18.7	18.7
	Very dissatisfied	6	4.5	5.6	24.3
	Neither satisfied nor dissatisfied	46	34.6	43.0	67.3
	Quite satisfied	22	16.5	20.6	87.9
	Very satisfied	13	9.8	12.1	100.0
	Total	107	80.5	100.0	
Lost	Lost	26	19.5		
Total		133	100.0		

**Table 7:** FSM Questionnaire.

5. Do You Avoid Sexual Intercourse because of Lumps in the Vagina (Bladder, Rectum, or Vagina)?					
		Frequency	Percentage	Percentage Valid	Cumulative Percentage
Valid	Always	32	24.1	29.9	29.9
	Frequently	13	9.8	12.1	42.1
	Sometimes	22	16.5	20.6	62.6
	Rarely	12	9.0	11.2	73.8
	Never	28	21.1	26.2	100.0
	Total	107	80.5	100.0	
Lost	Lost	26	19.5		
Total		133	100.0		

**Table 8:** Descriptive statistics of the quantitative variables.

Variable	N		Media	Medium	Typ. Dev.	Percentiles	
	Valid	Lost				25	75
1. Fear or anxiety about sex	110	23	1.21	0.00	1.626	0.00	3.00
2. Orgasm	110	23	1.63	1.00	1.561	0.00	3.00
3. Initial steps for a sexual encounter	110	23	0.63	0.00	0.975	0.00	1.00
4. Sexual satisfaction	107	26	3.02	3.00	1.228	3.00	4.00
5. Do you avoid sexual intercourse because of lumps in your vagina?	107	26	2.92	3.00	1.579	1.00	5.00

## Data Availability

Data not available.
